# A Review of Multi-Agent AI Systems for Biological and Clinical Data Analysis

**DOI:** 10.3390/mps9020033

**Published:** 2026-02-28

**Authors:** Jackson Spieser, Ali Balapour, Jarek Meller, Krushna C. Patra, Behrouz Shamsaei

**Affiliations:** 1 College of Medicine Cincinnati, University of Cincinnati, Cincinnati, OH 45267, USA; spiesejc@mail.uc.edu; 2 School of Computing and Analytics, Northern Kentucky University, Highland Heights, KY 41099, USA; balapoura1@nku.edu; 3 Department of Biostatistics, Health Informatics and Data Sciences, College of Medicine, University of Cincinnati, Cincinnati, OH 45267, USA; mellerj@ucmail.uc.edu; 4 Division of Biostatistics and Bioinformatics, Department of Environmental and Public Health Sciences, College of Medicine, University of Cincinnati, Cincinnati, OH 45267, USA; 5 Division of Biomedical Informatics, Cincinnati Children’s Hospital Medical Center, Cincinnati, OH 45229, USA; 6 Institute of Engineering and Technology, Faculty of Physics, Astronomy and Informatics, Nicolaus Copernicus University, 87-100 Torun, Poland; 7 Department of Computer Science, College of Engineering and Applied Sciences, University of Cincinnati, Cincinnati, OH 45219, USA; 8 Department of Cancer Biology, College of Medicine, University of Cincinnati, Cincinnati, OH 45267, USA; patraka@ucmail.uc.edu

**Keywords:** multi-agent systems, large language models (LLMs), biomedical AI, clinical decision support, orchestration frameworks, autonomous agents, AI safety, collaborative intelligence

## Abstract

This review evaluates the emerging paradigm of multi-agent systems (MASs) for biomedical and clinical data analysis, focusing on their ability to overcome the reasoning and reliability limitations of standalone large language models (LLMs). We synthesize findings from recent architectural frameworks, specifically LangGraph, CrewAI, and the Model Context Protocol (MCP), to examine how specialized agent teams divide labor, utilize precision tools, and cross-verify outputs. We find that MAS architectures yield significant performance gains in various domains: recent implementations improved oncology decision-making accuracy from 30.3% to 87.2% and reached a peak of 93.2% accuracy on USMLE-style benchmarks through simulated clinical evolution. In clinical trial matching, multi-agent frameworks achieved 87.3% accuracy and enhanced clinician screening efficiency by 42.6% (*p* < 0.001). However, we also highlight critical operational challenges, including an unreliability tax of 15–50× higher token consumption compared to standalone models and the risk of cascading errors where initial hallucinations are amplified across the agent collective. We conclude that while MAS enables a shift toward collaborative intelligence in biomedicine, its clinical and research adoption requires the development of deterministic orchestration and rigorous cost-utility frameworks to ensure safety and expert-centered oversight.

## 1. Introduction and Methodology

MAS, in which multiple autonomous agents collaborate as a team, are emerging as a powerful paradigm for biomedical and clinical data analysis [1,2]. Instead of relying on a single, all-purpose model, these systems orchestrate specialized AI agents, each with its own distinct knowledge base or tool, to perform complex tasks in a coordinated manner. A recent survey highlights this trend: LLM-based autonomous agents can achieve greater flexibility and robustness by dividing labor among sub-agents [1,3]. In various domains, genomics, electronic health records, imaging, and beyond, MAS approaches have already demonstrated improved performance, reliability, and explainability compared to single-agent methods. For example, collaborative agent teams have produced superior results on complex bioinformatics analyses and clinical decision support challenges where no single model excels [1,4]. Use cases range from automated information extraction in literature mining to multi-step clinical reasoning with tool-assisted LLMs [5,6].

Despite these promising developments, few domain-specific reviews examine how emerging MAS architectures are tailored for use in biomedicine. As AI tools become increasingly integrated into scientific and clinical workflows, synthesizing the current design patterns, orchestration strategies, and safety mechanisms is critical to guiding future research and deployment. At the same time, the rise of MAS introduces new challenges around communication protocols, shared memory, and guardrails for safety and accountability—all of which remain areas of active debate. For instance, while some researchers favor deterministic graph-based control structures to ensure reproducibility and auditability, others advocate for more flexible, emergent teamwork models that trade predictability for adaptability. These tensions underscore the need for a comprehensive, critical synthesis of the field.

Section 2 defines fundamental concepts that constitute an AI agent and describes frameworks for orchestrating multiple agents. We then survey the state-of-the-art applications in Section 3, covering broad basic science domains (genomics, drug discovery, etc.) and clinical domains (imaging, clinical trials, decision support). Section 4 discusses emerging opportunities and underexplored areas such as agent-assisted cancer research, data augmentation, and education. In Section 5, we summarize available platforms, toolkits, and benchmarks that support MAS development and evaluation. We then examine key challenges and future directions in Section 6, including reliability and verification of agent reasoning, scalability and efficiency issues, continual learning and adaptation, and ethical/regulatory considerations.

We argue that the orchestration layer—not merely the agent design itself—is increasingly the critical determinant of system performance, safety, and clinical relevance. By synthesizing recent literature, we aim to provide a timely and scholarly overview of how MAS can transform biomedical data analysis and what hurdles must be overcome for safe, effective deployment.

### Methods

We conducted a comprehensive literature search to identify relevant studies on multi-agent AI systems in biomedical and clinical domains. The search spanned multiple databases: PubMed, arXiv, ACM, and IEEE Xplore. The initial search strategy focused on the preceding six-year window (approximately 2019–2025) to capture the primary surge of interest in agentic systems and LLM-based approaches. However, given the rapid acceleration of the field, we extended our search window through 2026 via iterative manual screening of emerging preprints and recently published journal articles. This adaptive approach ensured the inclusion of core evidence, such as TxAgent, Agent Hospital, and MedAgentAudit, that represents the current state of the art but was published after the initial database queries.

Earlier studies were considered for inclusion only if they addressed foundational machine learning methods, ethics, or safety frameworks directly relevant to multi-agent systems. Additionally, regulatory frameworks from governing bodies, such as the FDA and HHS, were updated through March 2025 to reflect the most current guidelines on AI in medical devices. The search strategy employed various combinations of keywords, including “multi-agent systems”, “large language models (LLMs)”, “biomedical AI”, and “autonomous agents”, among others, to ensure broad coverage of the topic.

Identified articles underwent a screening and selection process based on predefined inclusion and exclusion criteria. To be included, a study needed to describe the implementation of a multi-agent AI system where multiple autonomous agents collaborated or coordinated to perform tasks. We excluded studies focusing on single-LLM systems, except where such a system was integrated into a larger multi-agent framework. After removing irrelevant or duplicate references, the remaining publications were assessed in full. We prioritized studies that reported performance or utility improvements of the multi-agent approach over single-agent baselines as evidence of the value added by agent collaboration. These selected studies form the basis for the structured synthesis presented in this review.

## 2. Definitions and Frameworks

### 2.1. Agent Definitions and Orchestration Frameworks

This section introduces the core concepts and orchestration frameworks that underpin modern MAS, providing the foundation for the case studies and analyses that follow. In artificial intelligence, an agent is commonly defined as an autonomous software entity that perceives its environment, makes decisions, and acts toward specific goals [1,3]. Modern formulations emphasize that an agent is an autonomous and collaborative entity, equipped with reasoning and communication capabilities, capable of dynamically interpreting contexts, orchestrating tools, and adapting behavior through memory and interaction across distributed systems [6]. Each agent possesses its own knowledge base and skills (e.g., domain expertise, task-specific tools) and can operate independently, adapting its actions based on observations. When multiple such agents work together, the result is an MAS. Compared to a single-agent setting, an MAS more realistically represents complex real-world scenarios involving multiple decision-makers or information sources [7,8]. Agents in an MAS typically have localized perceptions (each sees only part of the state) and must communicate or coordinate with others to achieve broader objectives. This distributed, team-based approach mirrors human teams, where each member has specialized roles and partial information, requiring collaboration to succeed.

MAS orchestration frameworks refer to the architectures and toolkits that facilitate coordination among multiple AI agents. These frameworks define how agents communicate, delegate tasks, and merge their results into a coherent solution [9,10]. Often, certain agents are designated as planners or managers who break down a user’s request into sub-tasks and assign them to specialist agents. For example, the HuggingGPT framework uses an LLM as a central controller agent to orchestrate numerous expert models (for vision, speech, etc.) as collaborative executors [11]. Similarly, Microsoft’s AutoGen system enables MAS conversations by allowing agents to spawn new agents and exchange messages to solve tasks cooperatively [12]. The open-source CAMEL toolkit provides templates for hierarchical agent societies (e.g., a MASter–worker structure where a manager agent plans high-level strategy, worker agents execute subtasks, and a reviewer agent checks outputs) [13].

Another popular open framework is LangGraph, which models a multi-agent system as a stateful graph, giving developers fine-grained control over execution paths. Agents and tools become nodes, and directed edges define the valid transitions between them [14]. This enables advanced control-flow patterns, conditional branches, loops, retries, and parallel paths, while retaining a strict definition of how the workflow may progress [15]. Unlike linear pipelines, LangGraph maintains a persistent state throughout the workflow: intermediate results, memory, and context are stored directly in the graph’s state object and passed deterministically across nodes. This ensures that agents operate with up-to-date information and prevents context loss during long-running interactions. These design choices make LangGraph particularly effective for structured, protocol-bound, or safety-critical applications where reproducibility and auditability matter. Because every transition is explicitly defined, LangGraph supports deterministic replay, transparent debugging, and insertion of human-approval nodes where appropriate.

CrewAI, by contrast, organizes a multi-agent system as a team of role-based agents—Planner, Executor, Researcher, Reviewer, and Critic—who collaborate through structured but adaptive message exchanges. Rather than enforcing a predefined graph, CrewAI leverages agent autonomy and role specialization to coordinate tasks dynamically based on the evolving problem state [14]. The framework provides high-level constructs (Agent, Task, Tool, Crew) and manages messaging, shared memory, and turn-taking automatically. Developers define roles and objectives, while the agents themselves decompose tasks, critique each other’s outputs, and refine results collaboratively. This approach is well-suited for creative, exploratory, or expertise-driven workflows where diversity of reasoning improves outcomes, such as itinerary design, literature synthesis, or complex multi-step reasoning.

These differences reflect two distinct orchestration philosophies: graph-driven determinism versus role-driven collaboration. Table 1 makes these distinctions explicit, comparing LangGraph and CrewAI across six operational dimensions: control flow, state handling, human-in-the-loop mechanisms, determinism, best-fit workloads, and limitations. In practice, LangGraph excels when workflows require strict branching logic, formal validation, or deterministic execution, while CrewAI shines when tasks benefit from adaptive teamwork and iterative critique.

Importantly, these paradigms are complementary rather than mutually exclusive. Modern systems increasingly employ hybrid designs in which LangGraph defines the global control flow while specific nodes delegate to a collaborative CrewAI team. This approach combines the determinism and safety guarantees of graph-centric execution with the flexibility and reasoning diversity of role-based collaboration. Thus, selecting between LangGraph and CrewAI (or combining them) depends on whether a workload demands protocol adherence, collaborative reasoning, or both.

**Figure 1:**
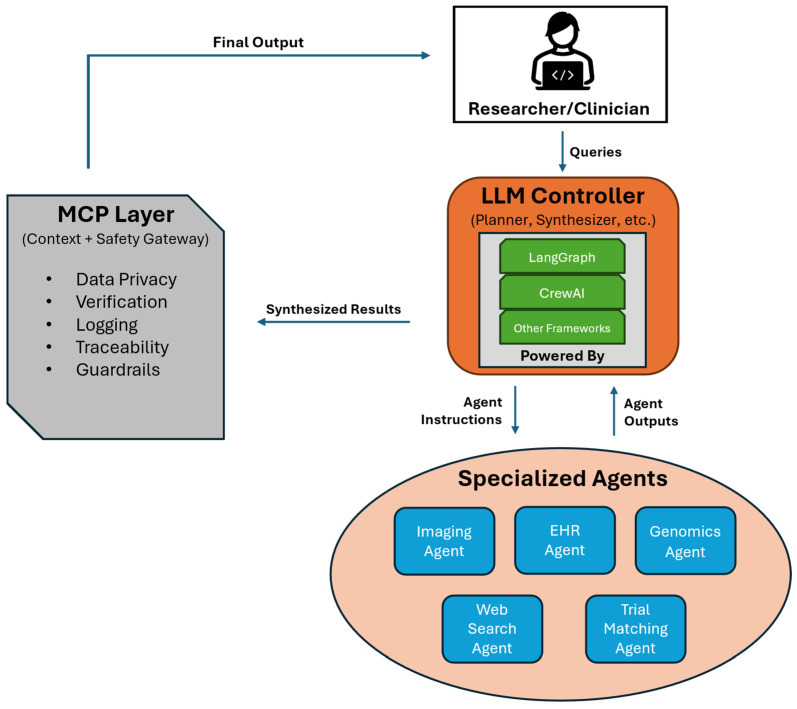
Conceptual architecture of a multi-agent system (MAS). A central controller orchestrates multiple specialized AI agents that interact with tools and external environments. Frameworks such as LangGraph and CrewAI support the orchestration of these agents by managing task delegation, communication, and memory. The MCP (Monitor–Control–Protect, discussed more in Section 6.1) layer acts as a context and safety gateway, implementing essential guardrails such as data privacy, verification, logging, and traceability to ensure secure and compliant agent collaboration.

In summary, MAS orchestration frameworks provide the infrastructure and design patterns (messaging systems, shared memory stores, standardized agent interfaces, etc.) to build systems where multiple AI agents collaborate and solve data-analysis problems more effectively than any individual agent alone [9,16]. This paradigm builds upon advances in LLM prompting and tool use. Techniques, such as ReAct, interleave logical Reasoning and concrete Acting steps by an LLM, demonstrating how language models can use external tools dynamically [17]. Similarly, methods such as Toolformer showed that LLMs can learn to call external APIs to aid in problem-solving [18]. These capabilities are often incorporated into agent frameworks, which could be a ReAct-style chain-of-thought (CoT) to decide when to query a database or run a simulation. MAS can also leverage strategies developed for single-agent prompting. For instance, an agent team can adopt a self-consistency approach where multiple agents independently work on the same problem and then vote or converge on an answer [19]. Agents can also implement a Tree-of-Thoughts strategy, collectively exploring different reasoning branches in parallel and exchanging information to decide on the best path forward [18,20]. Furthermore, various feedback and self-correction techniques have been introduced to improve LLM reliability, such as Reflexion and Self-Refine [21,22]. In this approach, a model iteratively critiques and revises its own answers. In an MAS context, such guardrails can be implemented by assigning a dedicated critic or reviewer agent to evaluate and correct the outputs of other agents [23,24]. By combining these approaches, hierarchical planning, tool use, parallel problem solving, and iterative self-correction, current frameworks are beginning to enable autonomous agent teams that are more robust and capable than any single AI agent. Figure 1 provides a high-level overview of a typical MAS architecture, highlighting the role of controller agents and orchestration frameworks such as LangGraph and CrewAI in managing specialized sub-agents.

### 2.2. Memory, Guardrails, and Communication Protocols

Effective MAS requires mechanisms for memory sharing, safety constraints, and structured inter-agent communication. Memory in an MAS context can refer to each individual agent’s internal context as well as a shared memory accessible to all agents. Modern agent frameworks often implement a shared knowledge store or blackboard that agents can read from and write to during collaboration [16]. This ensures that facts discovered by one agent (e.g., a relevant patient finding or an experimental result) persist and inform the others. For instance, agents working together could collectively build a knowledge graph or shared database of intermediate results as they progress through a task. Designing effective memory architecture is an active area of research. Challenges include how to retrieve relevant past information when needed and how to prevent context overload or forgetting important facts. Some systems use embedding-based vector memory to allow agents to recall long-term knowledge, while others use explicit storage of key-value records. As a simple example in biomedical NLP, one agent could store extracted patient information in a shared record so that another agent later can query “Has symptom X been noted?” instead of re-parsing the raw text. Shared memory also facilitates experience replay, where agents can learn from prior cases by referencing how similar problems were solved, improving continual learning (this is discussed further in Section 6.3).

Beyond memory, MAS must be engineered with guardrails to ensure safety and reliability. A team of agents autonomously generating and executing complex action sequences raises the risk of propagating errors or undesirable behaviors. One safeguard is to include a specialized monitoring or moderation agent that watches the agents’ interactions and intervenes if policies are violated (for example, halting the system if a medical-advice agent attempts an unsafe recommendation). Alignment techniques from single-agent LLMs, such as content filtering and reinforcement learning from human feedback (RLHF), can be extended to MAS settings by applying them to each agent’s outputs or decisions. Notably, in 2023, Google introduced an Agent-to-Agent (A2A) communication protocol as an open standard for LLM-based agents to exchange messages in a structured, secure manner [25,26]. Efforts such as A2A and related proposals for standardized Agent Communication Protocols aim to define common message formats (e.g., standardized JSON schemas or API calls) to enable interoperability and security in MAS ecosystems. However, these new protocols also introduce security considerations that must be addressed. An analysis of the A2A framework identified potential attack vectors (e.g., prompt injection, token reuse, agent impersonation) and emphasized the need for agent authentication and permission controls [27]. Ensuring secure and authenticated inter-agent communication will be vital so that a rogue agent cannot impersonate a trusted one or leak sensitive data. In general, MAS will require defense-in-depth: sandboxing of agent actions, well-defined scopes for each agent’s authority, and continuous monitoring for anomalous behaviors. We revisit safety and security issues in Section 6.1.

As these frameworks evolve toward increasingly autonomous and distributed architectures, these same communication and control mechanisms introduce new vectors for error propagation, bias amplification, or unsafe tool invocation. Accordingly, robust guardrails, spanning input validation, tool-permission control, and audit logging, are essential to ensure safe and transparent orchestration. These governance principles are discussed in greater depth in Section 6.4.

## 3. State of the Art in Biomedicine

MAS has rapidly gained traction as a strategy for tackling the complexity of biomedical data analysis and clinical decision-making. Researchers have already demonstrated that orchestrating multiple specialized agents can outperform single-model approaches on challenging tasks [28,29]. In this section, we survey representative state-of-the-art systems in both basic science and clinical domains. We organize the review into use-case categories, while noting that many systems span multiple categories. Throughout, we highlight how MAS designs are enabling new capabilities, from automating bioinformatics pipelines to running virtual clinical trials, and improving performance (e.g., accuracy, efficiency, interpretability) relative to prior methods.

### 3.1. Basic Science Applications

#### 3.1.1. Drug Discovery and Pharmacology

Drug discovery is a complex, multi-factorial process that can benefit from the division of labor offered by agentic workflows. Early examples of MAS in this domain show promise in integrating chemical databases, biological knowledge, and simulation tools to assist in identifying therapeutic targets or compounds. For instance, GPCR-Nexus is an MAS that focuses on G protein-coupled receptors (GPCRs), a large family of drug targets [30]. GPCR-Nexus employs an agentic OmniRAG approach: it orchestrates a team of agents that combine knowledge-graph traversal with retrieval-augmented text generation to answer pharmacological queries about GPCRs. One agent, a source planner, decomposes a user’s question into sub-queries for a literature search and a knowledge-graph lookup, while other agents execute those searches in parallel. A reviewer agent then filters and cross-checks the retrieved results for relevance and factual accuracy, and a synthesizer agent compiles a final answer with supporting evidence. By dividing labor (literature retrieval, structured data query, fact-checking, synthesis) among specialized agents, GPCR-Nexus produces context-rich, evidence-backed answers to questions that neither a conventional search engine nor a single LLM alone could easily handle. This case exemplifies how MAS can integrate structured knowledge bases with unstructured text mining to support drug discovery research by answering questions about receptor-ligand interactions, signaling pathways, and related clinical trials. Notably, the incorporation of a knowledge-graph agent helps reduce factual errors by grounding answers in curated databases, addressing a key limitation of standalone LLMs.

Another effort aimed at aiding therapeutic development is TxAgent (short for “Therapy Agent”). Introduced in 2025, TxAgent is an AI system that leverages multi-step reasoning and real-time knowledge retrieval across an extensive toolbox of domain-specific models (over 200 specialized tools in total) to support treatment discovery and optimization. In the prototype described by Gao et al., (2025), TxAgent can retrieve and synthesize evidence from multiple biomedical sources (e.g., drug-gene interaction databases, clinical guidelines, patient health records) and simulate the effects of drug combinations [31]. It assesses potential interactions between drugs and patient conditions, and iteratively refines treatment strategies using a reasoning loop. Although TxAgent is presented as a single composite agent, internally it functions as an MAS: separate sub-modules handle different tasks, such as chemical property prediction, pathway simulation, and literature scanning [31]. This highlights a continuum between a “single agent with many tools” and an “agent society”. In practice, a highly modular single agent (with distinct tool-using subroutines) begins to resemble a coordinated MAS. In benchmarked evaluations, TxAgent achieved 92.1% accuracy on DrugPC (open-ended drug reasoning), and 93.6%/93.7% on BrandPC/GenericPC, outperforming GPT-4o and other tool-use LLMs across 3168 drug reasoning tasks and 456 personalized treatment scenarios. These are benchmarked tasks; the manuscript provides limited clinical outcome evaluation, so we treat these as benchmarks rather than clinical endpoints. The system essentially serves as a virtual pharmacologist, and its design suggests a roadmap for MAS in drug discovery: one can imagine an expanded team where separate agents take on roles such as medicinal chemist designing novel compounds, toxicologist predicting safety profiles, clinical trial expert assessing trial feasibility, and so on, all coordinated to accelerate the drug development pipeline. While these benchmark results are promising, they do not yet represent established clinical endpoints, and the transition from therapeutic reasoning in simulated environments to real-world pharmacology remains an area for future investigation.

MAS in drug discovery is still in its infant stages, and most systems are prototypes or proofs-of-concept. Nonetheless, these examples illustrate the potential. By combining knowledge-driven agents (for biology and chemistry) with reasoning agents and simulation tools, MAS could, in the future, automate hypothesis generation for new drug targets, perform in silico screening of large compound libraries, and design optimal preclinical experiments. Moreover, MAS setups can naturally incorporate feedback loops with human scientists. For example, an agent team might propose a list of candidate molecules and then adapt its strategy based on a medicinal chemist’s feedback on which candidates are synthesizable. This collaborative human–AI approach aligns well with the iterative nature of pharmacological research.

#### 3.1.2. Bioinformatics and Multi-Omics Analysis

Bioinformatics was one of the first areas to embrace MAS, given the inherent complexity of biological data-processing pipelines. A current example is the BioMaster system by Su et al., 2025, an MAS framework for automated multi-omics workflows [32]. BioMaster integrates several specialized agents to plan and carry out genomic data analyses. For instance, a Planner Agent first decomposes a high-level bioinformatics task, such as “identify differentially expressed genes from these RNA-seq samples”, into a sequence of subtasks. Next, a Task-Executor Agent translates each subtask into concrete commands or API calls to bioinformatics software (e.g., running quality control, read alignment, variant calling, or statistical analysis). A dedicated Debug Agent monitors the pipeline for errors or suboptimal results (such as a file format issue or poor sequence coverage) and can intervene to adjust parameters or retry steps when needed [32]. Meanwhile, a Data Agent handles intermediate data caching and formatting between pipeline stages, and a Reviewer Agent validates final outputs, which includes checking whether the list of differentially expressed genes makes sense given known biology. BioMaster is equipped with dual retrieval-augmented generation modules: one consults domain-specific knowledge, namely method databases or prior experiments, to assist the Planner in choosing the best tools and parameters, and another helps the Debug Agent find solutions when a pipeline step fails. In benchmarking across 49 representative tasks spanning 18 omics modalities and 102 distinct bioinformatics tools, BioMaster completed substantially more analysis workflows than a baseline automated pipeline, especially on complex, multi-step analyses with interdependent tasks [32]. It has been demonstrated using both proprietary large LLMs and open-source models, indicating flexibility in the agent framework. Initial experiments suggest that MAS designs can bring robustness and adaptability to bioinformatics by uniting planning, execution, error-recovery, and validation agents; the system can handle data-processing challenges with minimal human intervention. This approach addresses inefficiencies in traditional pipelines, which often break when assumptions are violated, by enabling agents to detect and correct errors on the fly. It is important to note that Su et al. report relative improvements but do not specify exact percentage deltas; therefore, we treat this as illustrative, pending peer-reviewed, fully quantified results. As MAS frameworks similar to BioMaster mature, they could help democratize bioinformatics, allowing labs to input raw data and receive analyzed results with an intelligent agent team managing the entire workflow. This shift is further exemplified by the BioAgents system, which focuses on accessibility by leveraging small language models (SLMs) and retrieval-augmented generation to guide researchers through complex genomics tasks, even without extensive computational backgrounds [33]

#### 3.1.3. Cancer Biology

Cancer research and oncology are increasingly data-driven, and MAS are beginning to assist in these domains as well. One notable example is an AI-based “virtual tumor board” system for clinical decision-making in oncology. Ferber et al., (2025) developed an MAS that replicates a multi-disciplinary tumor board discussion by integrating multiple specialized agents for different data modalities and decision facets [34]. The system leverages GPT-4 as a core reasoning engine and incorporates specialized precision oncology tools, including vision transformers for detecting microsatellite instability (MSI) and mutations (KRAS, BRAF) from histopathology slides, MedSAM for radiological image segmentation, and web-based search tools like OncoKB and PubMed [34].

In a validation study involving 20 realistic multimodal patient cases focused on gastrointestinal oncology, the system was evaluated via a blinded manual review by four certified clinical experts. The MAS autonomously utilized appropriate tools with 87.5% accuracy (56 out of 64 required invocations) and reached correct clinical conclusions in 91.0% of assessed statements (223/245). Notably, the integrated agent improved decision-making “completeness”, or the ability to identify expert-predetermined interventions, from 30.3% (33/109) using GPT-4 alone to 87.2% (95/109) [34]. The agent also achieved a 94.0% helpfulness rate in addressing sub-questions and accurately cited guidelines 75.5% of the time. For comparison, state-of-the-art open-weights models like Llama-3 70B and Mixtral 8x7B registered significantly lower success rates of 39.1% and 7.8%, respectively [34].

These results demonstrate that the systematic integration of specialized diagnostic tools can effectively mitigate the genericity and reasoning bottlenecks of standalone LLMs in oncology. By delegating complex subtasks, such as lesion measurement via MedSAM or mutation prediction via vision transformers, to specialized agents, the MAS can synthesize a more comprehensive and evidence-backed treatment plan than a single model’s monologue. This marks a shift toward a paradigm where AI acts as a digital coordinator of diverse medical resources [28,29].

However, the transition to clinical practice requires addressing critical safety and scalability concerns. Despite the high accuracy, 2.4% (6/245) of the agent’s statements were flagged as potentially harmful, such as recommending suboptimal treatments, highlighting the persistent risk of errors in high-stakes environments. Furthermore, the study’s reliance on a small sample size (*n* = 20) and proprietary cloud-based models suggests that future research must prioritize large-scale prospective evaluations and the development of local, open-source architectures to ensure data privacy and clinical robustness across diverse hospital IT systems.

### 3.2. Clinical Applications

#### 3.2.1. Medical Imaging and Multimodal Diagnosis

Clinical diagnosis often requires integrating information from multiple sources, specifically physical exam findings, laboratory results, and medical images. MAS is naturally suited to such multimodal reasoning: different agents can specialize in processing distinct data types and then collaborate to form a comprehensive assessment. A recent study by Chen et al. (2025) provides a compelling example [35]. The authors developed an MAS framework for differential diagnosis, inspired by the multidisciplinary team meetings common in clinical practice. In their system, four AI “doctor” agents, each with a specialized knowledge focus or reasoning style, discuss a patient case, while a fifth agent acts as a supervisor to coordinate the conversation [35].

The multi-agent approach significantly outperformed single-model baselines in both initial diagnosis and follow-up consultations. On a dataset of 302 curated rare-disease cases (spanning categories such as abdominal surgery, cardiac, and genetic disorders), the MAS—referred to as the Multi-agent Conversation (MAC) framework in their paper—attained a 34.11% ‘most-likely diagnosis’ accuracy during the primary consultation, nearly doubling the performance of standalone GPT-4 (19.65%, *p* < 0.001) and GPT-3.5 (16.23%, *p* < 0.001). With complete information provided in follow-up consultations, the MAS accuracy for the most-likely diagnosis rose to 53.86%, significantly exceeding GPT-4 (37.86%, *p* = 0.010) and GPT-3.5 (29.36%, *p* < 0.001).

Furthermore, the ‘possible diagnoses’ accuracy reached 48.12% in primary consultations (compared to 34.55% for GPT-4, *p* = 0.001) and 67.88% in follow-ups (*p* = 0.042). The helpfulness rate for recommended further diagnostic tests reached 78.26%, a marked improvement over the GPT-4 baseline of 58.17% (*p* < 0.001). These results consistently exceeded not only standalone models but also advanced prompting variants. For instance, the MAC’s 34.11% primary accuracy beat CoT (27.81%), Self-Refine (32.01%), and Self-Consistency (32.45%) methods [35].

Qualitatively, the dialogues revealed that agents effectively introduced diverse perspectives, such as identifying rare syndromes or flagging lab abnormalities that contradicted specific hypotheses, leading to a more thorough analysis than a single model’s monologue. This MAS approach, mirroring an AI panel of consultants, offers a promising path toward making medical AI more reliable, transparent, and capable of reducing diagnostic oversight. However, token consumption for MAS reached over 6000 tokens compared to just 118 for standard input/output prompting. This indicates that a need to prioritize optimizing the efficiency of inter-agent dialogues to ensure these systems are both clinically transformative and economically viable for routine practice. Additionally, while the MAC framework provides a promising blueprint for diagnostic collaboration, further research is required to determine how such simulation-based successes translate to the unpredictable complexities of real-world clinical practice.

#### 3.2.2. Clinical Trials and Evidence Synthesis

Another area where MAS is making inroads is in clinical trial matching and evidence aggregation. Identifying suitable clinical trials for a patient, or conversely, finding patients for a trial, is essentially an exercise in complex criteria matching and data retrieval. This can be enhanced by deploying collaborative agents. A pioneering system in this realm is TrialGPT, which uses a trio of agents to match patients to clinical trials [36]. One agent, TrialGPT-Retrieval, first conducts a large-scale search to retrieve candidate trials from databases based on a patient’s profile; a second agent, TrialGPT-Matching, then evaluates the patient’s eligibility against each trial’s criteria using LLM-based reading and annotation of inclusion/exclusion criteria; finally, a third agent, TrialGPT-Ranking, assigns scores to rank the trials by suitability [36].

The system was then evaluated on three cohorts comprising 183 synthetic patients with over 75,000 trial eligibility annotations. In the initial pass, TrialGPT-Retrieval successfully recalled over 90% of relevant trials while filtering out most irrelevant ones, requiring less than 6% of the total collection to maintain this high recall. At the criterion level, manual evaluations of 1015 patient-criterion pairs demonstrated that the matching agent achieved an accuracy of 87.3%. Notably, the system achieved a precision of 90.1% and a recall of 87.9% in locating relevant supporting sentences in clinical notes. In the trial exclusion task, TrialGPT attained an AUROC of 0.7979, representing a 43.8% improvement over state-of-the-art baselines like the cross-encoder BioLinkBERT (AUROC: 0.6176).

A pilot user study revealed that clinicians assisted by TrialGPT were able to complete trial screening tasks 42.6% faster than those working without AI assistance (35.3 s vs. 61.5 s; *p* < 0.001) [36]. Ultimately, the high correlation between TrialGPT’s ranking scores and human judgment suggests that MAS has the potential to serve as a reliable cognitive assistant for complex, zero-shot patient-to-trial matching. By offering transparent, criterion-level explanations that allow clinicians to quickly verify AI decisions, this multi-agent architecture fosters the trust and scalability necessary to accelerate clinical research and broaden patient access to experimental therapies. However, as these evaluations were conducted on synthetic patient records, prospective studies are required to confirm whether these efficiency gains and matching accuracies hold within the disparate data structures of live hospital information systems [36].

Beyond matching patients to trials, MAS could assist in clinical trial design and evidence synthesis by forming a virtual committee to design a new trial protocol. For example, a Protocol-Writing Agent drafts a trial plan based on identified gaps in current research, a Critic Agent, encoded with ethical and feasibility rules, reviews the draft for potential issues and biases, and a Prior-Studies Agent searches for similar past trials to ensure novelty [37]. By iterating through these roles, the agent team can refine a trial proposal that a human investigator can then consider. This mirrors how human researchers brainstorm and critique protocols and could inspire more innovative trial designs. While still experimental, the idea has merit: AI agents excel at rapidly scanning large knowledge bases (all clinical trials done in a certain disease) to find what has or has not been tried. This can spark new ideas or help avoid duplication. Early steps in this direction are already evident. For instance, a research assistant agent named DORA was able to automatically draft biomedical research project proposals that were later refined by humans [38]. This suggests that similar agents could help assemble clinical trial protocols or grant applications. Likewise, MAS can serve as an evidence synthesizer for clinicians. One prototype system employs an Evidence Retrieval Agent to gather all published studies comparing certain treatments, and a Summary Agent to distill their findings into a concise report for a physician. In tests, this agent team could answer complex questions about comparative efficacy by synthesizing data across multiple studies, with higher accuracy and less hallucination than a single LLM working alone. As the body of medical literature continues to grow, such multi-agent evidence summation will be invaluable to support evidence-based practice.

In summary, MAS is proving valuable in navigating the maze of clinical trial data and medical literature. Agents for trial matching can systematically interpret both patient data and trial criteria with systems such as TrialGPT already attaining high accuracy, and agents for literature review can aggregate findings from numerous studies into coherent conclusions. By dividing tasks, searching, extracting, comparing, and summarizing, agents collaboratively produce comprehensive outputs that account for the full breadth of available evidence. This capability is increasingly crucial in medicine, where practitioners must keep up with rapidly evolving information. MAS can act as an ever-vigilant research assistant, ensuring that decisions (specifically enrolling a patient in a trial or choosing a therapy) are informed by the latest and most relevant evidence, something that human clinicians, burdened with information overload, would certainly welcome.

#### 3.2.3. Clinical Decision Support

One of the most impactful applications of MAS could turn out to be clinical decision support, or assisting physicians for diagnosis, treatment planning, and overall patient management. The complexity of real clinical cases, especially in fields such as internal medicine, often requires gathering disparate information, reasoning through possible diagnoses, consulting guidelines, and double-checking for errors or contraindications. MAS are naturally suited to handle these subtasks in parallel and provide a “second set of eyes” on each other’s work, thereby acting as a tireless medical assistant or consulting team for a physician.

A quantitatively robust example is the Agent Hospital framework, which utilizes a virtual hospital spanning 32 departments and 339 diseases [39]. The authors trained agents using 20,000 simulated patient encounters per task, evaluating them on a test set of 200 cases. A key innovation is the MedAgent-Zero architecture, which introduces an evolving agent architecture. MedAgent-Zero incorporates two memory systems: an experience base storing tens of thousands of simulated patient trajectories (symptoms → tests → diagnosis → outcome) and a case base containing curated medical Q&A exemplars. This enables the agent to learn from experience and refine clinical reasoning over time [39].

In simulated environments, the results suggest statistically significant performance gains through this evolutionary process. In cardiology, diagnostic accuracy for rheumatic heart disease improved from a 9% baseline (GPT-3.5) to 82% after treating approximately ten thousand simulated patients. On the MedQA (USMLE-style) benchmark, MedAgent-Zero achieved 92.22% accuracy, significantly outperforming the GPT-4o baseline (90.65%, *p* < 0.001) and standard CoT (90.42%). Ablation studies confirmed the necessity of both memory systems, as removing the experience base dropped accuracy to 91.36% [39]. While these results highlight that reasoning can be refined through simulation, the authors note that current improvements may follow a “scaling law” where gains slow as patient volume increases, suggesting an eventual performance ceiling. This demonstrates that the performance improvements arise specifically from the agent’s ability to accumulate experience and leverage structured case memory—confirming that multi-agent interaction, not only the base model, drives the accuracy gains in an experimental setting. Nevertheless, based on Miller’s Pyramid of Clinical Competence, it is critical to recognize that a 92.22% accuracy on MedQA benchmarks is a measure of the ‘Knows’ and ‘Knows How’ foundational levels of medical knowledge, rather than a surrogate for the ‘Shows How’ or ‘Does’ levels, which is required for dynamic, real-world patient interactions [40]. Recent systematic reviews of 39 medical AI benchmarks quantify a significant ‘knowledge-practice gap,’ finding that models achieving 84–90% on examinations often drop to 45–69% success rates when subjected to practice-based assessments that require diagnostic reasoning under uncertainty [41].

Moving on to another recently developed MAS, MDAgents (Kim et al., 2024), introduces an adaptive coordination framework that automatically assigns collaboration structures to a team of agents based on task complexity, mirroring the tiered referral processes used in clinical practice [42]. In evaluations across ten diverse medical benchmarks, including medical knowledge retrieval (MedQA, PubMedQA) and multimodal reasoning (Path-VQA, PMC-VQA), the framework achieved superior performance in seven out of ten tasks. In comparative benchmarking, MDAgents demonstrated a significant improvement of up to 4.2% (*p* < 0.05) over the best-performing prior methods, with specific accuracy gains reaching 5.2% on diagnostic reasoning datasets like DDXPlus. A key driver of this performance was the combination of moderator review and external medical knowledge retrieval (MedRAG), which resulted in an average accuracy boost of 11.8%. Beyond accuracy, the framework optimizes for efficiency. Static multi-agent settings often result in high computational costs (averaging 20.3 API calls), whereas the adaptive approach achieved peak accuracy using only 9.3 calls [42]. Furthermore, average inference times were found to scale with complexity, ranging from 14.7 s for straightforward “low” cases to 226 s for “high” complexity scenarios involving multidisciplinary care.

Looking ahead, the shift from static, text-only models to evolvable and adaptive multi-agent ecosystems marks a pivotal transition in medical artificial intelligence. By moving beyond “reading” medical literature to “practicing” within synthetic simulations similar to Agent Hospital, or dynamically assembling specialist teams via MDAgents, these systems begin to replicate the collaborative and iterative nature of real-world clinical decision-making. However, significant hurdles remain before hospital-wide deployment is feasible. Current frameworks are primarily limited by their focus on multiple-choice formats rather than the continuous, interactive narrative of patient-centered diagnostics. Furthermore, the risks of medical hallucinations and reasoning transparency require the integration of robust self-correction mechanisms and rule-based reward structures to ensure safety. Future research must prioritize the development of interactive systems that engage not only physicians but also patients and caregivers, incorporating regret-aware decision models to minimize diagnostic errors over time. Ultimately, MAS should be viewed as an assistive layer, or a digital ‘integrated care team’ designed to catch oversights and provide evidence-based suggestions. However, a clear distinction remains between simulation success and real-world bedside utility; prospective clinical trials are necessary to validate these systems as trustworthy collaborators in live patient care.

## 4. Opportunities and Underutilized Domains

While early successes are evident, there remain many domains in biomedicine and healthcare where MAS is underutilized or has yet to be explored. These represent exciting opportunities for future innovation. We highlight a few such domains and concepts below:

Meta-Science and Literature Curation: One intriguing use of agent orchestration is in scientific knowledge management itself. An example mentioned earlier is an “Awesome Bioagent Papers” repository autonomously maintained by an AI agent. Effectively, it is an agent that scans new publications and updates a curated list of important MAS research. Extending that idea, agent teams could continuously monitor the scientific literature, triage new papers, and update databases or summaries. For instance, one agent could scan preprint servers and PubMed daily for new papers in a specific field (say immunotherapy), another agent could extract key findings and methods, and a third agent could update a running literature review or knowledge graph with the new information. This kind of automated literature surveillance and synthesis goes beyond what any single model could do continuously. If realized, it would help researchers and clinicians stay up to date in rapidly moving fields. It also demonstrates a reflexive power of MAS: they can be used to improve themselves by discovering and aggregating the latest advancements in AI and biomedicine. Some early steps toward this vision are already underway. For example, an AI research assistant was able to draft research proposals and survey prior work with minimal human input [38]. In the coming years, we may see agent teams serving as “AI scientist” collaborators that generate hypotheses, design experiments, and analyze results alongside human scientists [43].

Rare Diseases and Personalized Medicine: These are areas where data are often sparse and expert knowledge is limited, making them perfect challenges for AI assistants. MAS could be set up as virtual tumor boards or case conferences for rare diseases, as described in Section 3.1.3 for oncology. Similarly, in personalized medicine, different agents could represent different knowledge realms about a patient—one agent specializing in the patient’s genomic data, another in their electronic health record, another in population health statistics, and together they would provide a comprehensive analysis to tailor treatment. Early research has shown that LLMs can generate synthetic patient data to augment real datasets for rare conditions [44]. A coordinated set of agents could take this further by generating entire synthetic patient cohorts for a rare disease (with one agent proposing plausible patient cases, another ensuring consistency with known disease biology, etc.), which could then be used to train and evaluate new diagnostic models. This is an opportunity to address data scarcity via multi-agent creativity under tight guardrails. Of course, safety and privacy would be paramount if agents are operating on real patient records; any such system would need strict oversight -and compliance with regulations. Nonetheless, the prospect of AI agent teams assisting with n-of-1 cases (the ultra-personalized scenario) is very compelling.

Biomedical Education and Training: MAS can also serve as educational tools. Consider a scenario where a medical student interacts with a team of AI “tutors”, perhaps a pathologist agent, a pharmacologist agent, and an ethics agent, who together teach the student by role-playing a clinical case. Each agent can provide expertise in its domain and correct the student (or each other) if a mistake is made. For example, the pathologist agent could describe microscope images and quiz the student on histology, the pharmacologist agent could ask dosing questions, and the ethics agent could pose a patient-consent dilemma. Such a system would provide a rich, interactive learning experience that mimics a multidisciplinary faculty, something a single AI tutor cannot easily achieve. Additionally, agent-based simulation environments (similar to Agent Hospital described earlier) can be used to train not only AI “doctors” but also human clinicians by simulating difficult or rare cases. Trainee doctors could practice managing virtual patients populated by AI agents that present realistic behaviors and symptoms [45,46]. This could accelerate training by exposing learners to a broader variety of scenarios than they might see during residency. MAS tutors have the added advantage of being available 24/7 and infinitely patient, allowing students to learn at their own pace. Although such applications are still experimental, they hint at a future in which medical (and scientific) education is supported by immersive simulations and responsive AI teaching teams.

In all these underutilized domains, the key idea is that MAS can tackle complexity and interdisciplinarity in ways single models cannot. By decomposing tasks and allowing agents to specialize (yet communicate), we open new frontiers for AI assistance: reading and summarizing a deluge of papers, hypothesizing about rare diseases, or training the next generation of clinicians and scientists. Capitalizing on these opportunities will require further research, as well as careful co-design with human users to ensure the AI agents truly augment human abilities in meaningful ways.

## 5. Platforms and Benchmarks

The ecosystem of platforms and benchmarks for MAS is rapidly maturing. Over the past two years, several open-source frameworks have been released to make building MAS more accessible. For example, LangChain and Hugging Face Transformers now include support for multi-agent dialogues and tool integration, allowing developers to script agent conversations with just a few lines of code. Microsoft has open-sourced Autogen (mentioned in Section 2.1) as a Python 3.10+ library, which provides high-level abstractions for spawning agents, managing their message exchanges, and incorporating new agents dynamically [12]. Similarly, academic groups have released research frameworks, namely CAMEL and MetaGPT. These provide templates for common MAS architectures (e.g., collaborative coding agents, conversational QA teams) [13]. These platforms handle much of the boilerplate (parallelizing agent runs, maintaining conversation histories, connecting to APIs) so that researchers can focus on agent behaviors and interactions. As a result, implementing a proof-of-concept multi-agent pipeline, which previously might have required custom threading and messaging code, is becoming far easier.

Alongside development frameworks, evaluation environments, and benchmarks are also being established. Researchers have created simulated worlds (virtual hospitals such as Agent Hospital) and game environments (Minecraft or Diplomacy) to systematically test MAS coordination, planning, and emergent behaviors. For instance, Arena and MAgent are multi-agent reinforcement learning platforms that have been adapted to LLM-based agents for analyzing how agents cooperate or compete. In the biomedical realm, there are efforts to construct realistic evaluation scenarios. For example, a mock clinical Turing test where an agent team must triage patients in an emergency-room simulation, or a Bioinformatics Grand Challenge where agents compete to correctly annotate a genomic dataset. Recently, Zhang et al., (2024) introduced Agent-SafetyBench, a suite of tests to evaluate whether agents respect certain safety and ethical rules in medical and general contexts [27]. In this benchmark, MAS are challenged with scenarios (making treatment recommendations under specific constraints) and scored on rule adherence. Such community benchmarks will be crucial for comparing different approaches and tracking progress. Early results from Agent-SafetyBench have already helped identify weaknesses in current systems’ guardrails, guiding researchers toward better training and oversight mechanisms.

Beyond task performance, diagnostic tools for MAS are emerging. Developers have begun creating “AgentOps” dashboards that monitor agent interactions, resource usage, and errors in real time (analogous to MLOps for model deployment). For example, an AgentOps interface might show that the Medication-Recommender Agent in a clinic support system has had a recent spike in corrections from the Safety-Checker Agent, prompting a developer to investigate a possible drift in the recommender’s behavior. Visualization tools are being built to map out conversation trees among agents or to replay multi-agent trajectories for analysis [12,13,14,15,45,46]. All of these contribute to a better understanding of how agent teams operate and where improvements are needed.

In sum, the ecosystem of platforms and benchmarks for MAS is quickly expanding. There are now robust frameworks to build and run agent systems, rich environments to test them in simulated clinical and biological scenarios, and an expanding knowledge base of case studies that illustrate design patterns. This virtuous cycle of shared tools and evaluation standards will accelerate progress. Early adopters in biotech and healthcare are already experimenting with these frameworks to automate tasks once thought too complex for AI, and as more results and code are shared, development becomes easier and more standardized. The stage is being set for MAS to move from concept to practice in biomedicine, supported by a community infrastructure for development and benchmarking.

## 6. Challenges and Future Directions

MAS in biomedicine is a fast-moving and promising field, but significant challenges remain in scaling these systems and ensuring they operate safely and effectively. In this section, we discuss key hurdles and research directions under several themes: reliability and verification, scalability and efficiency, continual learning and adaptation, and ethical/regulatory considerations.

### 6.1. Reliability, Verification, and Safety

Ensuring that a team of AI agents works reliably is more complex than verifying a single model’s output. With multiple agents interacting, new failure modes emerge. Errors can propagate or even be amplified if agents take each other’s faulty outputs at face value. For instance, a retrieval agent might fetch irrelevant or misleading data, and a reasoning agent could incorporate it into a diagnosis, leading to an incorrect conclusion. Therefore, techniques for verification and validation are paramount in MAS [24,27]. One basic strategy is to incorporate redundancy, having multiple agents independently attempt the same task and then compare results. This is analogous to the self-consistency method for single LLMs (multiple reasoning paths followed, then a majority vote taken); in a multi-agent setting, each agent (or agent subgroup) provides a second opinion [19]. If two diagnostic agents disagree, that flags uncertainty for human review or triggers the system to gather more information. Another strategy is built-in cross-checks: agents explicitly evaluating each other’s outputs. For example, after a Question-Answering Agent proposes an answer, a Reviewer Agent can assess its factual accuracy and coherence, similar to the Reflexion approach (where an agent self-critiques and iterates), but here implemented as a separate agent [23,24]. The system can iterate in a loop (plan → answer → review → refine) until a consensus or confidence threshold is reached. Empirically, this kind of reviewer setup has been shown to reduce hallucinations in LLM-generated answers [47,48]. Multiple studies report that letting an agent analyze and criticize an initial draft leads to more factual and internally consistent responses.

Formal verification methods, common in safety-critical software, are also being considered for MAS. One could imagine specifying logical constraints or rules that the agents must follow (e.g., “No treatment recommendation should violate known contraindications”) and using a symbolic reasoning agent to verify that all agent actions adhere to these constraints. While true formal proofs may be infeasible for complex language-based behaviors, even partial formalization, such as checklists or rule-based audits performed by an agent, can help catch errors. For instance, Zhang et al., (2024) introduced Agent-SafetyBench to evaluate whether agents respect certain safety rules in their reasoning [27]. MAS could employ an internal safety-auditor agent during operation, essentially running SafetyBench-style tests on the fly (e.g., scanning agents’ messages for disallowed content or scanning decision steps for rule violations) and intervening when needed. Already, expanded attack surfaces and emergent risky behaviors have been observed in autonomous agent experiments [49,50,51]. For example, research by meta-AI groups showed that a sufficiently empowered LLM-based agent could autonomously orchestrate multi-step cyberattacks under certain conditions, and instances of agents deviating from user intent (even into harmful actions) have been documented when goals are misaligned. These findings underscore the importance of rigorous safety testing and constraints before deploying MAS in high-stakes biomedical settings. Developing verification frameworks that combine statistical testing, symbolic checks, and adversarial simulations will be an important area of future work. Ultimately, building trust in MAS will require clear evidence that these systems perform reliably under real-world conditions and that there are safeguards to catch and correct errors before harm can occur.

The Model Context Protocol (MCP) has emerged as a key infrastructure to ensure that AI agents can access external tools and data (e.g., databases, enterprise APIs, clinical records) in a safe, transparent, and regulated manner. MCP is an open standard interface that defines a unified, bi-directional communication channel between AI models and external resources. In essence, MCP follows a client–server architecture in which an AI agent (client) never connects to sensitive systems directly; instead, it issues requests to a trusted MCP server which mediates every action. The MCP server acts as an intelligent broker between the agent and the data source or service: it enforces predefined policies and schemas on all requests, then queries the actual external system on the agent’s behalf. This design ensures principle-of-least-privilege access. In other words, the agent can only see or do what the MCP’s policy allows, and any out-of-scope request is rejected before it touches a secure database or API. All context exchanged is structured and constrained: MCP uses JSON-RPC 2.0 messages with strict schemas for inputs and outputs. By eliminating free form prompts in favor of structured JSON payloads, MCP reduces the risk of prompt injection or unexpected data leakage, since every query and response must conform to an expected schema and type. Crucially, MCP enables real-time policy enforcement: every tool invocation or data query from an agent can be evaluated against security and privacy rules before execution, with the MCP server blocking or flagging any disallowed actions automatically [52].

For example, an organization might set an MCP policy that permits an agent to read a patient’s medication records but forbids writing back to the health database or accessing identifiable patient info unless a higher authorization is provided—the MCP middleware will simply deny any request that violates these constraints, keeping the AI within approved bounds. This not only guards privacy and safety, but also makes the system more explainable and auditable. The MCP server maintains an immutable audit log of every interaction, recording which agent asked for what data, which policies were applied, and what response was returned, thereby providing full transparency into the agent’s activities. Such visibility is vital in domains such as healthcare, where accountability and trust are paramount. By enforcing standardized request formats, access controls (e.g., role-based permissions, user consent requirements), and comprehensive logging, MCP acts as a safety layer—essentially a “secure USB port” through which AI agents can plug into critical data sources without breaching compliance or confidentiality. Researchers and industry stakeholders are actively extending MCP for domain-specific needs (for instance, exploring a Healthcare MCP profile aligned with HL7/FHIR standards) so that next-generation AI collaborators in medicine and other fields remain bounded, interpretable, and lawful in their use of real-world data [53]. Finally, it is important to note MCP is only a communication standard; thus, intra-system errors from agent reasoning are still a possibility.

### 6.2. Scalability and Efficiency

Current MASs, especially those based on large language models, face practical challenges in scalability and computational efficiency. Running several LLM agents in parallel or in long sequential dialogues can be resource-intensive in terms of CPU/GPU usage and memory. As the number of agents grows, communication overhead can also grow combinatorially, a phenomenon sometimes called “agent flurry,” where agents produce a torrent of messages that can slow down the system. Ensuring that MAS remains responsive and cost-effective when scaled up is therefore a key technical challenge.

One issue is the context length and memory: If each agent needs the full history of the conversation or the entire patient record, the token usage can explode. Solutions being explored include selective context sharing (agents only receive relevant excerpts of the state) and hierarchical communication structures (agents communicate through a central hub or in clusters, rather than all-to-all). For example, a hierarchy might involve a manager agent that condenses the team’s intermediate findings and passes summaries to other agents, rather than every agent seeing every message raw. This was implicit in some frameworks, such as CAMEL, and future systems will likely formalize such hierarchies to curb communication blow-up.

Another challenge is optimizing agent specialization to avoid unnecessary redundancy. If too many agents overlap in functionality, the system wastes time and computing resources on duplicate efforts. Techniques such as agent role differentiation, perhaps via specialized fine-tuning for each agent’s subtask, or using smaller models for simpler agents can make the system leaner. For instance, one might use a large LLM for a complex reasoning agent but a smaller, cheaper model for a routine data-extraction agent that feeds it. Early systems such as MDAgents began to explore this by dynamically deciding whether a task needs a “group” or can be handled by one agent [39].

The architectural trade-offs of MAS become most apparent when evaluating the ‘efficiency-accuracy curve.’ While early evaluations such as the MAC framework reported a 50-fold increase in token consumption (6000 vs. 118) for a roughly 15% absolute accuracy gain, recent large-scale clinical evidence suggests this ‘accuracy premium’ is vital in high-stakes domains [35]. For instance, in complex medical coding tasks where diagnostic specificity is tied to financial and legal outcomes, a RAG-enhanced agentic system demonstrated superior performance to human providers; expert reviewers favored the AI’s accuracy in 447 instances compared to 277 for provider-assigned codes (*p* < 0.001) [54]. Furthermore, the system was preferred for its specificity in 509 cases versus 181 for human providers (*p* < 0.001), illustrating that the high token expenditure of agentic loops is economically justified when the cost of diagnostic or administrative error exceeds the marginal cost of increased computing.

Latency is also a concern for clinical applications: doctors need answers fast. Parallelizing agent operations and allowing asynchronous processing can help. In a well-designed MAS, not all agents need to operate in lockstep; one agent could be searching literature while another parses patient data, and they synchronize once both have results. Achieving this kind of concurrency will require careful orchestration but could yield major speedups.

From a software engineering perspective, orchestration infrastructure will need to become more robust to handle dozens of agents over long uptimes. This includes agent state management (keeping track of each agent’s context when they might be paused and resumed), error handling (if one agent crashes or produces gibberish, the system should catch it and perhaps restart that agent or query a backup agent), and scaling across machines (distributing agents over a cluster when one machine is not enough). Cloud providers are already looking into “agent hubs” that can dynamically allocate resources to agents as demand fluctuates.

In summary, managing the scalability of clinical MAS requires a transition from individual token optimization to enterprise-level cost-utility analysis. While the architectural overhead of agentic orchestration is significant, the socioeconomic burden of diagnostic errors, which is estimated to affect up to 1.8% of GDP in developed economies, provides a strong argument for the “accuracy premium” offered by collaborative designs [55]. For patients with rare, undiagnosed, or complex diseases, MAS has the potential to mitigate the heavy financial burden offsetting computational costs through the reduction of redundant secondary testing and acute care utilization [41]. Future systems must therefore utilize dynamic orchestration to selectively deploy agentic teams based on task complexity, ensuring that resource-intensive multi-agent deliberation is reserved for high-stakes clinical scenarios where the cost of error exceeds the marginal cost of compute [38,56].

### 6.3. Continual Learning and Adaptation

Most current MAS rely on foundation models that have a fixed knowledge cutoff and do not automatically update as new data arrives. In fast-moving biomedical fields, this is a significant limitation. New research findings, drug approvals, or clinical guidelines emerge constantly. For MAS to remain useful over time, they will need the ability to continuously learn and adapt. This challenge spans multiple aspects: keeping the knowledge base up to date, learning from new cases, and evolving agent strategies.

One promising approach is to implement an online learning loop where agents automatically fine-tune or adjust based on feedback. For example, after each deployment or interaction, an MAS could perform a brief review: Did the agents achieve the desired outcome? Were there any mistakes identified by human users or a critic agent? These could be fed into a replay buffer or used to update the agents using reinforcement learning with feedback or few-shot learning on recent corrections. Prior work on lifelong learning for LLM-based agents provides a roadmap here [47]. Zheng et al. (2025) outlined strategies for incorporating lifelong learning into agent architecture by dividing the system into a perception module (to handle new modalities), a memory module (to accumulate evolving knowledge), and an action module (to adapt behaviors) [47]. In an MAS context, one can imagine certain agents devoted to monitoring performance and triggering updates to other agents as needed.

Memory mechanisms will play a central role in continual learning. A shared memory store can act as a growing knowledge base that agents query to get up-to-date information. Rather than retraining models from scratch, agents might consult an ever-expanding knowledge graph of medical facts or a vector database of past case embeddings. In Section 2.2, we discussed how memory agents can cache intermediate results; extending that concept, memory agents can cache new lessons learned. For instance, if an MAS encounters a novel drug–drug interaction in one case (perhaps flagged by a human doctor during deployment), it could record that in the shared knowledge base so that future consultations avoid the same oversight.

Another aspect is adapting to shifts in the environment. Clinical practice in one hospital may differ from another; patient populations differ; even the preferred communication style of clinicians may vary. MAS potentially needs a period of local tuning to the specific setting. This might be achieved by allowing the agents to observe and participate in discussions with human clinicians, gradually calibrating their suggestions to the local standards of care. One could designate a “shadow” phase where the agent team only observes decisions and provides commentary (which is checked but not acted on), and based on discrepancies with actual decisions, the agents adjust their decision thresholds or reasoning patterns. However, continual learning in deployed AI systems carries risks: models can forget old knowledge when fine-tuned on new data (catastrophic forgetting), or they can drift in undesirable ways (if new data is biased or if adversarial inputs are encountered). Ensuring stable learning is therefore important. Techniques similar to experience replay (re-training on a mix of old and new cases) and periodic evaluation against fixed benchmarks can help detect when an agent’s performance on prior knowledge starts degrading.

Lifelong learning for biomedical MAS is still largely uncharted territory. Encouragingly, the modular nature of MAS may aid continual learning since knowledge can be compartmentalized. For example, a drug-interaction agent could be updated independently of a symptom-checker agent if their interface is maintained. This modular update ability could prevent the entire system from needing frequent full retraining. Recent surveys on lifelong LLM agents highlight emerging trends. Notably, modular retraining, meta-learning to learn how to learn, and the use of external memory to mitigate forgetting [47]. We anticipate these ideas will be progressively incorporated into biomedical agent teams. Ultimately, the vision is for MAS to never stop learning and continually grow its medical expertise as new data and knowledge become available, similar to how a human professional keeps up through continuing education.

### 6.4. Ethics, Regulation, and Trust

The deployment of MAS in biomedicine raises important ethical and regulatory considerations. Many of these are extensions of concerns with single-model AI, but some issues are amplified or uniquely manifested in MAS. Here we outline a few key considerations and the path forward to address them:

**Accountability and Transparency:** When an AI team makes a recommendation or decision, who is accountable if something goes wrong? With multiple agents contributing, it can be unclear which agent’s action or which interaction led to an error. This “many hands” problem complicates assigning responsibility. From a regulatory perspective, it may be necessary to log detailed transcripts of agent dialogues and decision paths so that any failures can be audited after the fact [57]. Ensuring transparency will be crucial for trust. This could involve providing human users with traceable explanations. An agent team’s final report could include a summary of their internal discussion (which symptoms each agent prioritized, which sources they consulted, and how they reached consensus). Such explanations might be more comprehensive than the reasoning of a single model, since different viewpoints were considered. Research in XAI (explainable AI) is beginning to explore how to generate user-friendly explanations from multi-agent processes [58].

**Bias and Fairness:** Combining multiple agents does not automatically cancel out biases in the underlying models or data. In fact, there is a risk that agents could reinforce each other’s biases (if, say, a lead agent has a bias and other agents trust its conclusions). Careful evaluation of biases, whether concerning race, gender, socioeconomic status, or other sensitive attributes, is needed before clinical deployment. Techniques such as having a “devil’s advocate” agent specifically question decisions from a fairness perspective could be one safeguard. Regulators expect evidence that MASs have been tested for equitable performance across diverse patient groups [59]. Just as clinical trials demand subgroup analyses, AI agent teams may need to demonstrate that their recommendations do not systematically underperform or overtly disadvantage any demographic.

**Data Privacy:** MASs often intensively share and process data, which raises privacy concerns. If patient data is used across agents, the system must comply with health information privacy laws (HIPAA in the US, GDPR in Europe, etc.). Each agent’s access should be limited to the minimum necessary data for its function, also known as the data minimization principle. Communication between agents should ideally be encrypted and occur on secure local networks when dealing with identifiable health data. There is also a question of data provenance: if an agent pulls information from an external source (online database), that source must be trustworthy and compliant with data use agreements. Ongoing work on federated learning and secure multi-party computation might inform how agents can operate on sensitive data without pooling it centrally [60]. For example, agents could pass computed insights (“protein X is highly expressed”) rather than raw data (the entire gene expression matrix), reducing exposure of raw patient data.

**Regulatory Approval:** MAS tools intended for clinical use will likely fall under regulatory scrutiny (e.g., the FDA’s software as a medical device framework). Regulators will need to consider not just the performance of the system, but its failure modes, update mechanisms, and transparency. One challenge is that MAS can exhibit emergent behaviors not readily predictable from their components (as has been observed in some social simulations). This can make validation tricky; traditional static testing might not capture an agent team’s behavior in all scenarios. Regulatory science may need to embrace new validation methods, such as simulation-based stress testing, which involves throwing thousands of randomized scenarios at the agent team and analyzing outcomes [58,60]. There may also be a move toward conditional approvals or post-market surveillance: approving an AI assistant for use with the condition that it logs all recommendations and outcomes, so any systematic issues can be caught early. The FDA has already signaled interest in adaptive AI that learns over time, proposing monitoring approaches to ensure safety is maintained during algorithm updates [58]. MAS that adapt (as in Section 6.3) will need to fit into such frameworks, perhaps with agent-specific validation when one component changes.

**Trust and Acceptance:** Finally, for MAS to be adopted, human practitioners and patients must trust it. This goes beyond raw accuracy: it involves human factors. Doctors will need to feel that the AI team is a wise colleague rather than a mysterious black box. Building that trust may require incorporating clinicians into the development loop so they can give feedback on agents’ behavior, as well as a user-friendly design of the agent interface that presents information in natural, helpful ways. Studies on physician attitudes toward AI have found that clear explanations and the ability to veto or override AI suggestions are important for acceptance [59,60]. MAS should be designed so that users remain in ultimate control: the agents propose, but the human disposes. Involving ethics boards and patient advocacy groups early in the deployment of such systems can also surface concerns and shape guidelines for appropriate use. For example, there may be contexts where the AI should not intervene or where a human must always double-check, such as end-of-life care decisions, which might be flagged for human-only deliberation due to their value-laden nature.

In conclusion, while MAS holds great promise, realizing that promise responsibly will require careful attention to ethical, legal, and social implications. The complexity that gives these systems power also makes them challenging to govern. Ongoing research in AI ethics, along with emerging standards (e.g., IEEE’s AI ethics standards, FDA/EMA guidelines on clinical AI), will need to be applied and likely extended for multi-agent scenarios. With thoughtful design and oversight, MAS can be deployed in ways that enhance healthcare delivery while upholding the core principles of medicine: beneficence, non-maleficence, autonomy, and justice.

Collectively, these ethical domains highlight that responsible MAS deployment depends not only on ethical principles but also on *technical and procedural guardrails*, concrete mechanisms that ensure these values are enforced during design, operation, and oversight. These controls operationalize ethics and regulation, ensuring that the values discussed above manifest in system design, deployment, and oversight. Table 2 consolidates the essential guardrails required for the ethical and regulatory deployment of multi-agent systems in biomedicine, drawing on NIST AI RMF (2023), ISO 14971:2019, IEC 62304, IMDRF SaMD guidance, and HIPAA/GDPR frameworks. Each domain addresses a distinct failure risk: accountability and transparency mitigate the “many-hands” problem; bias and fairness guard against cumulative model skew; data and privacy governance prevent unauthorized PHI exposure; safety and oversight mechanisms restrain unverified autonomy; evidence and auditability ensure provenance; and regulatory alignment sustains compliance across the MAS lifecycle.

Modern frameworks increasingly embed these safeguards within orchestration logic. Immutable logs enable end-to-end traceability, fairness-auditor agents interrogate outputs for subgroup bias, and approval gates halt unsafe actions before execution. Collectively, these measures operationalize the core biomedical ethics of beneficence, non-maleficence, autonomy, and justice, while aligning MAS design with reproducibility and verification requirements defined for Software-as-a-Medical-Device (SaMD) systems under ISO 14971 and IEC 62304.

The adoption of standardized guardrails reframes ethics as an engineering discipline, allowing regulators and clinicians to inspect not only what an agent decided but also how that decision was formed. Yet such rigor introduces trade-offs: stringent permissions and human review may reduce system agility, whereas relaxed constraints risk ethical or legal failure. Future MAS research should emphasize adaptive governance, dynamic risk scoring, continuous post-market surveillance, and participatory co-design with clinicians and ethicists, to preserve equilibrium between autonomy and accountability. In practice, these layered controls form the connective tissue between technical reliability and institutional trust, ensuring that multi-agent intelligence evolves within clear ethical and regulatory boundaries rather than ahead of them. For clinicians and data scientists, this structured guardrail framework clarifies which controls are mandatory (e.g., PHI encryption, audit trails) versus adaptive (e.g., fairness-auditing cadence), bridging the gap between conceptual ethics and operational practice.

## 7. Discussion

**Critical Perspective on MAS Efficacy:** The promising results reviewed above must be interpreted with caution. A notable bias in the current literature (and thus in our review) is the focus on success stories—many MAS studies highlight impressive outcomes, whereas failures or neutral results often go unreported. This publication bias can lead to an overly optimistic view of multi-agent approaches. Recent research has begun to critically examine these systems and suggests that collaboration is not a panacea. In fact, simple ensembling methods (majority voting) may account for most of the performance gains initially attributed to complex agent interactions [66]. In other words, some multi-agent setups appear to outperform single large models not due to teamwork, but because averaging out errors yields more robust answers. Such findings are a cautionary reminder that MAS must be rigorously evaluated rather than assumed superior by default. Researchers and clinicians should not assume that adding more agents automatically yields better outcomes. Going forward, more ablation studies and negative results need to be published to identify when and how specific agent team compositions truly add value versus when they introduce unnecessary complexity.

Beyond reporting bias, a fundamental ‘knowledge-practice gap’ remains a bottleneck for clinical adoption of MAS [41]. While systems like Agent Hospital excel at simulated, text-based USMLE benchmarks, these scores primarily validate the ‘Knows’ and ‘Knows How’ foundational levels of Miller’s Pyramid of Clinical Competence, which effectively demonstrate a readiness to learn rather than a readiness to practice [39,40,67]. A 2025 systematic review of 39 medical LLM benchmarks quantified a 19.3 percentage point drop in success rates when moving from examination-based pattern recognition (84–90%) to practice-based assessments (45–69%) [41]. This disparity suggests that current models suffer from “inflexible reasoning,” or the Einstellung effect, where they rely on pattern matching from training data rather than engaging in the adaptive, threshold-based decision-making required at the bedside [41,68]. The implication is a dangerous “miscalibrated overconfidence”: agents produce fluent, high-confidence outputs even when accuracy in life-critical safety assessments drops as low as 40–50% [68]. Ultimately, the bigger picture indicates that exam scores are a misleading proxy for clinical readiness; the transition from “AI scientist” to “AI clinician” requires moving beyond static facts toward high-fidelity, practice-oriented frameworks that mandate rigorous human-in-the-loop oversight to bridge the gap between theoretical knowledge and bedside utility [41,67].

**Practical Limitations and Costs:** Our review also reveals several practical challenges that temper the excitement around MAS. One major concern is the computational overhead and complexity of coordination. Orchestrating multiple large language models is resource-intensive: context must be shared back-and-forth between agents, and they often engage in lengthy dialogues. This dramatically increases runtime and cost, as each agent’s inference and communication incurs additional latency and token usage [69]. A solution that works in a controlled research demo might thus be too slow or expensive for real clinical settings. MAS developers are exploring ways to mitigate this overhead. For example, using smaller specialized models for simple sub-tasks, or limiting inter-agent communication to only essential information. However, the fact remains that parallelism comes with significant cost. Another limitation is the risk of cascading errors. With multiple agents in a system, a faulty output from one agent can mislead others and propagate or amplify the mistake throughout the system. We described cases where a “reasoner” agent might base its decision on misinformation retrieved by another agent, leading the entire team to a wrong conclusion. Robust cross-check mechanisms (such as reviewer or auditor agents) have been proposed to catch such errors, but not all MAS implementations include them. This underscores the need for strong verification and validation frameworks tailored to MAS. Encouragingly, researchers have recently begun systematically auditing multi-agent medical systems to catalog their failure modes. For example, a 2025 study developed a taxonomy of collaborative failure patterns, including flawed consensus driven by shared model biases, suppression of correct minority opinions, ineffective discussion dynamics, and critical information loss during synthesis [70]. Developing such taxonomies of how and why MAS fail will help guide the design of safer agent collaborations. It also reinforces that high aggregate accuracy alone is insufficient: we need methods to ensure each agent team’s reasoning process is sound and verifiable, not just the final answer.

**Figure 2:**
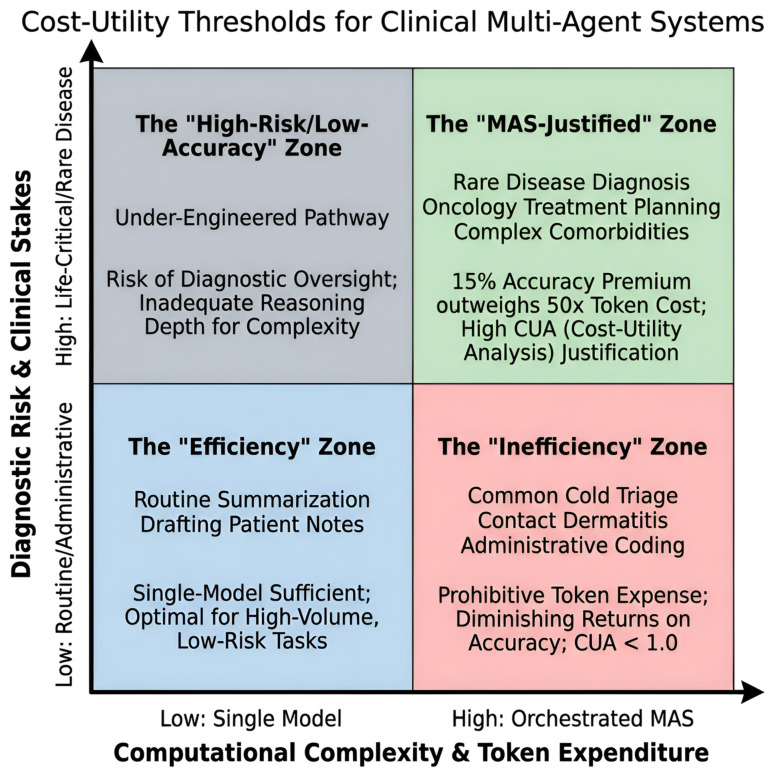
Clinical Cost-Utility Thresholds for MAS. Matrix classifying scenarios where a ~15% absolute accuracy gain justifies a 50-fold increase in token consumption. High-stakes tasks in the MAS-Justified Zone (top right) leverage improved specificity to mitigate diagnostic error costs ($7850 to $2397/case). Low-risk tasks in the Inefficiency Zone (bottom right) demonstrate diminishing returns (CUA < 1.0) where high-compute communication is economically prohibitive. Single models remain optimal for the Efficiency Zone (bottom left), while the High-Risk/Low-Accuracy Zone (top left) warns of diagnostic oversights from insufficient reasoning depth [35,41,54,55,56,71,72].

**High-Stakes vs. Low-Stakes Utility—Practical Guidelines:** To navigate the trade-off where MAS may consume 50 times more tokens than standalone models to achieve a roughly 15% absolute accuracy gain, we propose a classification framework based on Cost-Utility Analysis (CUA). CUA is an economic evaluation that compares the relative costs and outcomes (utilities) of different interventions, allowing for a standardized assessment of whether high-expenditure systems provide sufficient clinical value, such as gains in Quality-Adjusted Life Years (QALYs) [71]. This categorization defines where the ‘accuracy premium’ of orchestrated agents is justified. See Figure 2.

- **High-Stakes Scenarios (Justified):** In domains such as oncology or the identification of rare, fatal conditions, a 15% improvement in diagnostic precision has the potential to be transformative. The systemic burden of diagnostic safety lapses is estimated at 1.8% of GDP in OECD countries [55]. For patients with suspected rare diseases, uncoordinated clinical investigations typically incur costs 7.6-fold higher than matched controls [41]. In these high-morbidity settings, the sub-dollar cost of additional tokens is negligible compared to the thousands of dollars saved by mitigating redundant secondary testing and acute care utilization. Recent evaluations indicate that structured agentic panels can reduce complex diagnostic expenditures from approximately $7850 to $2397 while significantly increasing specificity over human specialists [54,55].
- **Low-Stakes Scenarios (Prohibitive):** Conversely, for routine, high-volume tasks such as the triage of the common cold, contact dermatitis, or administrative chart summaries, a 50-fold cost increase provides diminishing returns. In these low-risk environments, the marginal gains do not offset the increased latency, API costs, or computational footprint [41,55]. For such tasks, standalone models remain the preferred economic choice, as the clinical utility of multi-agent deliberation does not warrant the “unreliability tax” of excessive agent communication.

**Integration, Data, and Compliance Challenges:** Beyond algorithmic performance, there are pragmatic issues of integrating MAS into real biomedical workflows. Interoperability is a key hurdle: agents must interface with diverse data sources, databases, and software tools used in biomedicine. Ensuring seamless communication and data exchange among agents, and between agents and existing hospital IT systems, is non-trivial. Many MAS prototypes remain confined to the lab and have never been plugged into electronic health record systems or laboratory information management systems. Additionally, data privacy and security impose strict constraints. Healthcare data are highly sensitive, so any clinical MAS will need to maintain compliance with regulations like HIPAA, employ robust encryption, and enforce role-based access controls for patient information. Designing agent communication such that only the minimum necessary patient data are shared between agents is a complex requirement (to avoid inadvertently pooling or leaking sensitive information). There are early efforts to address these issues; for instance, the proposed Model Context Protocol (MCP) mediates agent access to external data with fine-grained security rules, acting as an intelligent broker that filters queries and enforces policies [52,53,64]. Such technical solutions for safe data handling are actively being explored to make multi-agent workflows secure and compliant by design.

Regulatory approval is another looming challenge. If an MAS is intended as a clinical decision support tool, it may qualify as a medical device subject to FDA or EMA oversight. Regulators may ask: how do we validate an AI team that can change its own workflow on the fly? Traditional validation methods, which assume a fixed algorithm, may not suffice for adaptive, learning agent teams. Indeed, the FDA has acknowledged that its traditional paradigm for device regulation was not designed for continuously learning AI/ML systems [62,63,73]. New approaches to validation and quality assurance have been suggested, such as simulation-based stress testing of MAS behaviors and continuous post-market monitoring of their performance in the field. In short, the real-world deployment of MAS will require not just improving AI accuracy, but also surmounting engineering, data governance, and regulatory barriers that lie outside the scope of typical model development. Success will depend on coordinated efforts to resolve these systems-level issues so that multi-agent intelligence can be safely and seamlessly integrated into clinical practice.

## 8. Future Directions

Despite the challenges discussed, the emergence of MAS in biomedical research and healthcare carries profound potential. If current obstacles can be overcome, MAS could enable a new level of AI-assisted discovery and clinical care. For example, one can imagine AI “teammates” that integrate knowledge across genomics, proteomics, and clinical data silos, or that continuously survey and summarize the flood of new biomedical literature. Realizing this vision will require the community to pursue several key directions in research and development. Below, we outline a few priority areas for future work:

- **Rigorous Real-World Evaluation**: The MAS community should move beyond purely benchmark-driven experiments and conduct real-world deployment studies. Pilot implementations, for instance, testing a multi-agent system as a virtual tumor board in an oncology department, or as a decision-support assistant in live hospital workflows, would be invaluable for assessing true utility and reliability [35]. Such trials will help determine whether MAS can genuinely improve outcomes or efficiency in practice, and they will reveal unanticipated failure modes and human–AI interaction challenges that are not evident from offline simulations.
- **Technical Robustness and Efficiency**: Continued research is needed to improve the reliability, scalability, and efficiency of MAS. This includes incorporating redundancy and consensus mechanisms as standard practice to improve accuracy. Communication protocols must evolve toward ‘value-aware’ orchestration, where agentic depth is dynamically scaled based on the Cost-Utility Analysis (CUA) of the task at hand. Rather than applying a uniform agentic reasoning to all queries, future MAS should autonomously reserve high-compute multi-agent deliberation for high-stakes clinical scenarios, such as working up rare or complex disease variants, while utilizing leaner single-agent pathways for routine administrative triage [66]. Methods for safe continual learning are also important so that agent teams can update their knowledge bases as medical knowledge evolves, without compromising prior validated performance. Additionally, there is a clear need for better agent observability and debugging tools. For example, dashboards or immutable logs that allow developers and regulators to inspect how decisions were reached by the agent collective. Improving transparency and auditability of MAS reasoning will build confidence that these systems are working as intended [69]. Together, advances in these areas will determine whether MAS can evolve from exciting demos into trusted, production-grade systems.
- **Human-Centered Design and Governance**: Finally, the development of MAS should be guided by human-centered principles. Domain experts, such as clinicians, biomedical researchers, and pharmacologists, must be involved in co-designing multi-agent solutions for their workflows. Human oversight should remain integral to MAS deployments: studies consistently indicate that keeping doctors and scientists involved is essential for reviewing AI outputs, correcting errors, and making the ultimate decisions. Features that facilitate this oversight and build user trust are crucial. For example, developers should include explanation modules (an interface that clearly explains the team’s reasoning in human-understandable terms) and implement fail-safe triggers that defer to human judgment when the system’s uncertainty is high or when ethical boundaries might be crossed [74]. Importantly, MAS should be positioned as assistive tools that enhance human expertise, rather than autonomous entities that supplant it. By designing for transparency, control, and accountability from the start, we can ensure these AI agent teams are adopted as dependable collaborators in medicine and science. With diligent interdisciplinary effort, spanning AI research, software engineering, clinical evaluation, and ethics, MAS can mature into a transformative asset for biomedicine.

## 9. Conclusions

MAS are charting a path toward biomedical AI systems that collaborate in a manner analogous to human teams by combining specialized skills, cross-checking each other, and jointly tackling problems beyond the scope of any single model. From the examples surveyed, this approach offers unique advantages. By breaking down complex tasks into collaborative subtasks and instituting internal checks-and-balances, multi-agent designs address many shortcomings of standalone LLMs. We see early evidence that they can reduce oversights, catch inconsistencies, and provide clearer rationale through inter-agent dialogue. Prototype systems in drug discovery, bioinformatics, and clinical decision support have already illustrated these benefits, in some cases achieving results that rival or exceed prior single-model methods. Nonetheless, the transition from ‘AI scientist’ to ‘AI clinician’ requires more than just high benchmark scores; it demands a fundamental bridging of the knowledge-practice gap. As we have shown, the true value of MAS lies not in replacing human judgment, but in providing a high-fidelity ‘safety component’ that justifies its computational cost by preventing the massive socioeconomic burden of diagnostic error. By aligning agentic reasoning with the tiered competencies of Miller’s Pyramid, we can ensure that these systems are validated for real-world bedside utility rather than just theoretical examination performance.

However, substantial progress and rigorous validation are still needed before such systems can be widely adopted in real-world biomedical settings. The coming years will be critical for moving from promising prototypes to clinically and scientifically validated tools. We expect to see many more prospective clinical trials and deployment studies of MAS to determine where these systems add value and where human oversight remains essential. Such examples include evaluating an AI agent team as a virtual physician assistant in a hospital department or integrating an agent-driven bioinformatics pipeline in a pharma research project. Studies similar to the ones just described will provide invaluable feedback on utility, failure modes, and best practices for human–AI collaboration. They will also help earn the trust of clinicians and scientists by clearly demonstrating the conditions under which agent teams improve outcomes versus where they might introduce new risks or create more work.

This paper has several limitations. First, given the quick evolution of the field, new results (especially preprints) have likely emerged after our literature search cutoff, so some relevant developments may not be captured. Second, many of the studies we summarized evaluate MAS on benchmark datasets or simulated scenarios rather than in live clinical environments, which means their reported success may not directly translate to real-world settings where unpredictability is higher. Third, heterogeneity in evaluation settings, differences in datasets, agent configurations, and outcome metrics makes it difficult to directly compare performance across systems. Finally, our focus on LLM-based MAS means that other paradigms (e.g., multi-agent reinforcement learning or symbolic agent systems) are only partially represented; our conclusions should be viewed as reflective of the current LLM-centric state of the field. These limitations suggest caution in over-generalizing the findings. We advise readers to stay up to date with the latest research as this domain continues to mature rapidly.

In conclusion, it bears emphasizing that MAS are not about replacing human researchers or clinicians, but about augmenting and assisting them. When designed with care, AI agent teams can handle information overload, perform tedious or complex multi-step analyses, and offer second opinions or suggestions that a busy human might miss. This enables human experts to focus more on empathy in patient care, insight in experimental design, or ethical judgment in difficult decisions. If the challenges outlined above can be addressed, MAS could become a vital asset in understanding biology and treating diseases. We envision a new collaborative partnership between humans and intelligent software agents for the advancement of science and health. The long-term vision is AI scientists working together with human scientists, each complementing the other’s strengths and pushing the frontiers of biomedicine in ways neither could achieve alone.

## Figures and Tables

**Table 1:** Comparison of LangGraph and CrewAI.

Dimension	LangGraph	CrewAI
Control Flow	Explicit, developer-defined state machine. Nodes are agents/tools; edges are deterministic transitions. Supports branching, loops, retries, parallelism.	Emergent role-based delegation. Planner, Executor, Reviewer coordinate dynamically. No fixed global graph: flow adapts to agent decisions.
State Handling	Centralized, persistent graph state passed between nodes; memory is first-class and scoped to workflow steps.	Distributed state via shared crew memory and message passing. Less centralized; context emerges from dialogue rather than a single state object.
Human-in-the-loop	Native support through approval nodes, gating steps, and enforced checkpoints.	Supported through reviewer/critic roles or human task overrides, but less formally structured.
Determinism	High. Given the same inputs and settings, execution is reproducible and replayable.	Medium. Agent decisions depend on role behavior, LLM variability, and message contents; reproducibility possible but less guaranteed.
Best-fit Workloads	Safety-critical, protocolized workflows; regulated pipelines; workflows needing strict branching logic, validation, compliance, or deterministic replay.	Creative ideation, collaborative reasoning, decomposition and critique tasks, research workflows, expert-team simulations, open-ended problem solving.
Limitations	More upfront design effort: large graphs can become complex; less flexible for exploratory tasks.	Less deterministic; harder to enforce strict protocols; role interactions can become noisy or redundant if not carefully designed.

**Table 2:** Core Guardrails for Ethical and Responsible MAS Deployment in Biomedicine.

Guardrail Area	Core Control Mechanism	Biomedical Implementation Example
Accountability & Transparency	Persistent, tamper-evident decision logging; structured explainability layers that expose intermediate agent reasoning; human-approval checkpoints for consequential actions.	Append-only audit trails capturing prompts, model versions, and tool outputs; clinician-readable summaries of agent deliberations before final recommendations.
Bias & Fairness	Continuous subgroup-performance auditing; counterargument or “devil’s-advocate” agents to challenge consensus; diversity of model sources to avoid correlated bias.	Periodic bias stress-tests across sex, race, and socioeconomic strata; fairness-review agents questioning diagnostic or treatment disparities.
Data & Privacy Governance	Principle of least privilege; automatic de-identification; end-to-end encryption; retrieval allow-lists; local-only data residency where possible.	Role-scoped access to de-identified EHR records; encrypted inter-agent channels; content filters that block PHI leakage during retrieval or tool use.
Safety & Human Oversight	Clinical rule-packs for contraindications and dosage safety; approval gates for high-risk or off-label actions; fallback to deterministic single-agent mode on anomalies.	Embedded drug–drug interaction checks; dual sign-off for therapeutic orders; circuit-breakers that halt unsafe multi-agent cascades.
Evidence & Auditability	Provenance enforcement (“no-evidence, no-claim”); automated citation extraction; confidence-linked references; replayable decision provenance.	Each recommendation linked to guideline ID, DOI, or PMID; confidence scores with evidence hyperlinks; reproducible workflow re-runs for audits.
Regulatory Alignment & Trust	Mapping of system functions to SaMD risk classes; integration of ISO 14971/IEC 62304 life-cycle controls; post-market drift monitoring and human-in-the-loop review.	Documented verification and change-control plans; real-time performance dashboards; clinician feedback loops during adaptive model updates

Each guardrail area represents a critical governance layer that translates ethical and regulatory principles into concrete system controls. These categories synthesize best practices and international standards relevant to biomedical MAS, including the NIST AI Risk Management Framework [61], ISO 14971 [62], IEC 62304 [63], IMDRF SaMD N41 [64], the HIPAA Privacy Rule [65], and Zhang et al., Agent-SafetyBench [27].

## Data Availability

Corresponding data is available at http://gpcr-nexus.org (accessed on 28 September 2025).
